# Increased serum level of alpha-2 macroglobulin and its production by B-lymphocytes in chronic lymphocytic leukemia

**DOI:** 10.3389/fimmu.2022.953644

**Published:** 2022-09-02

**Authors:** Regina Michelis, Lama Milhem, Evleen Galouk, Galia Stemer, Ariel Aviv, Tamar Tadmor, Mona Shehadeh, Lev Shvidel, Masad Barhoum, Andrei Braester

**Affiliations:** ^1^ The Institute for Medical Research, Galilee Medical Center, Nahariya, Israel; ^2^ Azrieli Faculty of Medicine, Bar Ilan University, Safed, Israel; ^3^ Institute of Hematology, Galilee Medical Center, Nahariya, Israel; ^4^ Department of Hematology, Emek Medical Center, Afula, Israel; ^5^ Hematology Unit, Bnai Zion Medical Center, Haifa, Israel; ^6^ The Ruth and Bruce Rappaport Faculty of Medicine, Technion, Haifa, Israel; ^7^ Biochemistry Laboratory, Galilee Medical Center, Nahariya, Israel; ^8^ Hematology Institute, Kaplan Medical Center, Rehovot, Israel; ^9^ Faculty of Medicine, Hebrew University, Jerusalem, Israel

**Keywords:** alpha-2-macroglobulin (A2M), B-lymphocytes, chronic lymphocytic leukemia, complement system, classical pathway of complement

## Abstract

Chronic lymphocytic leukemia (CLL), the most common adult’s leukemia in the western world, is caused in 95% of the cases by uncontrolled proliferation of monoclonal B-lymphocytes. The complement system in CLL is chronically activated at a low level *via* the classical pathway (CP). This chronic activation is induced by IgG-hexamers, which are formed after binding to alpha-2-macroglobulin (A2M). The study investigated for the first time the serum levels of A2M in CLL patients, their association with the disease severity, and A2M production by the malignant B-lymphocytes. Blood samples were collected from 65 CLL patients and 30 normal controls (NC) subjects, and used for quantifications of the A2M levels, the complement activation marker (sC5b-9), the complement components C2, C3 and C4, and clinical biochemistry and hematology parameters. The production of A2M was studied in B-lymphocytes isolated from blood samples as well as in CLL and non-CLL cell lines.The serum A2M levels were significantly higher in CLL patients vs NCs, showing values of 3.62 ± 0.22 and 1.97 ± 0.10 mg/ml, respectively. Within the CLL group, A2M levels correlated significantly with the disease stage, with sC5b-9, and with clinical indicators of the disease severity. Increased A2M production was showed in three out of four CLL B-lymphocytic lines that were studied, as compared to non-CLL lines, to a non-lymphocytic line, and to blood-derived primary B-lymphocytes. A2M production was further increased both in primary cells and in the CLL cell-line after incubation with CLL sera, compared to NC sera. This study shows for the first time that serum A2M levels in CLL are significantly increased, likely due to A2M production by the malignant B-lymphocytes, and are correlated with the disease severity and with chronic complement activation. The moderate change in A2M production after incubation with NC sera *in-vitro* supports the hypothesis that inhibition of excess A2M production can be achieved, and that this may potentially down-regulate the IgG-hexamerization and the resulting chronic CP activation. This may also help restore complement system activity, and eventually improve complement activity and immunotherapy outcomes in CLL.

## 1 Introduction

Alpha-2-Macroglobulin (A2M), the largest major non-immunoglobulin protein in human plasma, is a broad-spectrum protease inhibitor that can inhibit all proteinase families, including serine-, cysteine-, aspartic- and metalloproteinases (1). Each A2M molecule contains a macroglobulin domain, a thioester (TE) containing domain and a receptor-binding domain (2). Its protease-inhibitory functions are executed *via* a conformational change that traps the attacking protease after binding to a TE motif, GCGEQ, also called “the bait” within the A2M protein (3). Similar to other members of the thioester-containing proteins (TEPs) family, named after their active site that functions by forming covalent bonds with specific molecular targets, A2M plays an important role in clearance of proteases from the circulation, regulation of fibrinolysis, coagulation and complement activation (3). A2M also participates, with IgG molecules, in formation of immune complexes that generate aggregated/hexameric structures (A2M-IgG-hexamers) and activate the classical pathway (CP) of the complement system in patients with Chronic Lymphocytic Leukemia (CLL) (4).

A2M is an acute-phase protein, produced primarily by the liver and lungs, and secreted into the bloodstream and extracellular environments (5). It is also produced locally by macrophages, fibroblasts, and epithelial cells (5). A2M is formed by the assembly of four 182kDa subunits into two disulfide-linked dimers, which non-covalently associate to complete the tetrameric structure of A2M (1). However, several studies showed that A2M is present in the circulation in either a dimeric or tetrameric form (6, 7).

Mutations in the A2M gene play a role in the pathogenesis of Alzheimer’s disease, Parkinson’s disease, and prostate cancer (1), while increased serum concentrations were shown to be associated with nephrotic syndrome, diabetes (type 1 and type 2), chronic liver disease and acute lymphoblastic leukemia (8–10). In patients with CLL, A2M has rarely been studied, and the serum levels of A2M have not yet been reported.

CLL is the most common leukemia, comprising 25-30% of all adult leukemia patients in the western world. It affects the B-type lymphocytes in the bone marrow in 95% of the patients, and is characterized by increased numbers of monoclonal B-lymphocytes (>5x10^3^/µl) that express specific antigens (CD5, CD19, CD20, CD23) on their surface. A2M is involved in the chronic CP activation in CLL (4), which decreases the CP activity in these patients. This decrease in CP activity compromises the CP dependent mechanisms activated by the immunotherapeutic drugs, such as the complement dependent cytotoxicity (CDC), and may eventually affect the outcomes of the immunotherapy. Thus, the aims of the study were to measure A2M levels in CLL sera in various stages of the disease and to assess its production by the malignant B-lymphocytes.

## 2 Materials and methods

### 2.1 Subjects

Blood samples were collected from 65 un-treated (naïve) CLL patients and 30 normal (non-malignant) controls (NC). As CLL patients are normally ~69 years old, many of them also suffer from chronic diseases such as type 2 Diabetes mellitus and/or hypertension, which were not used as exclusion criteria. Accordingly, the NC group included subjects who were matched with the CLL group for gender, chronic diseases and age (as possible). NC exclusion: any type of malignancy in the past or present, and any type of severe infection or hospitalization within the past 6 months. Plasma and sera were separated and frozen at -80˚C. The study was approved by the Helsinki Committee (Institutional Review Board) of Galilee Medical Center (Nahariya, Israel), and all subjects signed informed consent.

### 2.2 Measurement of A2M levels

#### 2.2.1 ELISA

To measure the A2M and C2 levels, commercial ELISA kits were used (human A2M and human C2 ELISA kits, both from Elabscience), according to the manufacturers’ instructions. The results were measured using an ELISA reader (CARIOSKAN LUX, Thermo scientific).

### 2.3 Analysis of A2M production by B-lymphocytes

#### 2.3.1 Cell culture

The following CLL B-lymphocytes cell lines were used: MEC-2 (CSC-C0511 from Creative-Bioarray, USA), JVM-2 (CRL-3002 from American Type Culture Collection, USA/ATCC), JVM-13 (CRL-3003 from ATCC) and HG-3 (ACC 765 from DSMZ-German Collection of Microorganisms and Cell Cultures GmbH, Germany). All cell lines were cultured at 37°C in a humidified 5% CO_2_ atmosphere. JVM-2, JVM-13 and HG-3 were cultured with RPMI 1640 medium (ATCC) and MEC-2 in IMDM medium (Biological Industries). The medium for MEC-2 and HG-3 was supplemented with 10% heat-inactivated fetal bovine serum (hi FBS, Biological Industries, Israel) and non-hi FBS for JVM-2 and JVM-13. The monocytic cell line THP-1 (TIB-202 from ATCC) as well as two B-lymphocytes cell lines from non-CLL origin (from patients with Diffuse Large B-cell Lymphoma (DLBCL): SU-DHL-4 and SU-DHL-5 (CRL-2957 and CRL-2958, respectively, from ATCC) were used as controls. THP-1 cells were cultured in RPMI 1640 medium and 20% hi FBS, while SU-DHL-4 and SU-DHL-5 cells were cultured in RPMI 1640 medium and 10% non-hi FBS. All growth media were supplemented with 0.1% penicillin-streptomycin (ATCC) and the growth media of MEC-2 and JVM-2 were also supplemented with 1% glutamine (Biological Industries, except for THP-1), and all cell lines were cultured for <8 passages.

#### 2.3.2 Isolation of primary B-lymphocytes from peripheral blood

Primary B-lymphocytes were isolated from peripheral blood as described previously (11) except that the isolation procedure was performed under sterile conditions. Blood was obtained from patients and NC subjects in EDTA-containing tubes and used immediately for separation of peripheral blood mononuclear cells (PBMCs) by Lymphoprep (Axis-shield) density gradient centrifugation in SepMate™ PBMC Isolation Tubes (STEMCELL Technologies Inc.). Cell numbers were counted in a hemocytometer and 10^7^ PBMCs were further used for B-lymphocyte isolation, based on negative selection, using magnetic-activated cell sorting (MACS) bead Isolation kits (Miltenyi Biotec). B-lymphocytes from CLL and NC subjects were isolated with B-Cell Isolation B-CLL Kit (130–103–466) and B Cell Isolation Kit II (130–091–151), respectively. In order to validate the purity of separated primary B-cells, they were studied by flow-cytometry, using CD19 and CD20 antibodies. Cell suspensions (10^5^ cells) were washed twice with PBS, and stained for 10 min with the fluorescent antibodies anti-CD19-Phycoerythrin Cyanin 7 (CD19-PC7, Beckman) and anti-CD20-Allophycocyanin (CD20-APC, protein-tech), that bind to B-lymphocytes. All incubations were performed at room temperature (RT), in the dark. The staining of the cells was assessed by a Flow cytometer (Navios, Beckman Coulter).

#### 2.3.3 A2M production in B-lymphocytes

Cells of the CLL B-lymphocyte cell lines were centrifuged at 120g for 7min at RT, re-suspended in fresh culture medium at a concentration of 20,000 cells/100µl, and incubated in triplicates in a 96 well plate, in the 37°C incubator. After 12-72 hrs, depending on the cell type (12 hrs for lines MEC-2, HG-3, JVM-13, SU-DHL-4 and SU-DHL-5; 24 hrs for the THP-1 line; 72 hrs for the JVM-2 line), the triplicates were combined, centrifuged at 5000 g for 5 min at RT to remove the cells, and the supernatants were stored at -20°C until the A2M analysis (by ELISA) was performed. The incubation time for each cell type were selected after evaluation of several time points (data not shown).

In parallel, cells were centrifuged at 120g for 7min at RT and re-suspended in fresh medium at a concentration of 20,000 cells/80µl. Sera of CLL patients or NC subjects, with known A2M levels, were added to a final concentration of 20%, and 100µl of the mixture was incubated in triplicates in a 96 well plate, and processed as described above. A2M levels were measured in the cell’s medium, and the production of A2M by the cells was calculated as the change in A2M amount (ΔA2M in µg) after subtracting the level contributed by the serum, and the levels produced by the cells without any added serum.

For examination of A2M production in primary B-lymphocytes, B-cells isolated from peripheral blood of CLL patients and NC subjects were centrifuged at 120g for 7min at RT, re-suspended in fresh culture medium (RPMI 1640 and 10% non-hi FBS) at a concentration of 20,000 cells/100µl or 20,000 cells/80µl and incubated with or without 20% human serum as described above for the cell-line cells. After 24 hrs the triplicates were combined, centrifuged at 5000g for 5min at RT to remove the cells and the supernatants were frozen for later A2M analysis (by ELISA). The ΔA2M was calculated, in µg, as described above.

### 2.4 Statistical analysis

Data were analyzed by unpaired, two tailed, student t-test. Data from the *in-vitro* experiments were analyzed by Mann-Whitney tests (two tailed), as appropriate. P<0.05 was considered significant.

## 3 Results

### 3.1 Characteristics of the subjects’ groups

The Clinical parameters of patients and NCs are shown in **Table 1**. As expected, all the hematological parameters in CLL patients were significantly different from the values in the NC group, so that WBC, lymphocyte counts, and lymphocyte percentage were higher while RBC, Hb, and platelets counts were lower, relative to NC. The mean patient’s age was higher relative to the NC group, however A2M levels were not influenced by age (**Supplementary Figure S1**). One liver function indicator, ALT, was significantly decreased in the patients, although within the normal range. Two lipid profile parameters, the HDL and total cholesterol levels, were significantly decreased in the patients, as reported previously (12). No significant differences were observed in all other parameters.

**Table 1:** Characteristics of the subjects’ groups.

Clinical parameter	CLL patients	Normal controls	p-value
n	65	30	
Gender (male/female)	41/24	19/11	
Age (years)	67.6 ± 1.3*	59.6 ± 1.9	**0.001**
ALP U/L (40-150)	87.6 ± 6.7	74.2 ± 3.6	0.105
ALT U/L (<55)	20.0 ± 1.4*	27.9 ± 2.1	**0.002**
AST U/L (5-34)	23.3 ± 1.0	24.4 ± 0.8	0.502
Cholesterol mg/dl (<200)	161.5 ± 6.3*	196.9 ± 8.4	**0.001**
Triglycerides mg/dl (<150)	151.0 ± 10.7	147.8 ± 17.9	0.932
HDL mg/dl (>40)	35.1 ± 1.9*	47.3 ± 2.8	**<0.001**
LDL mg/dl (<100)	96.4 ± 6.1*	120.8 ± 7.9	**0.016**
Non-HDL chol. mg/dl (<130)	125.7 ± 6.5*	149.6 ± 8.3	**0.023**
Serum C3 mg/dl (82-185)	127.8 ± 6.1	119.5 ± 3.6	0.320
Serum C4 mg/dl (15-53)	26.5 ± 1.6	31.5 ± 1.5	0.052
WBC x10e^3^/µl (4-10)	49.5 ± 8.8*	6.9 ± 0.3	**0.002**
Lymph. abs x10e^3^/µl (1.5-8)	44.9 ± 9.6*	2.2 ± 0.1	**0.001**
Lymph. % (19-48)	69.0 ± 2.5*	30.9 ± 1.2	**<0.001**
Platelets x 10e^3^/µl (130-400)	159.2 ± 9.2*	243.4 ± 9.3	**<0.001**
RBC x 10e^6^/µl (4-5.5)	4.3 ± 0.1*	4.8 ± 0.1	**0.003**
Hb g/dl (13-18)	12.9 ± 0.3*	14.1 ± 0.3	**0.006**

The results of subjects’ characteristics are shown as mean ± SEM. ALP, alkaline phosphatase; AST, aspartate transaminase; ALT, alanine transaminase; HDL, high density lipoprotein; LDL, low-density lipoprotein; non-HDL Chol., Non-HDL Cholesterol; C3, complement component 3; C4, complement component 4; WBC, white blood cells; Lymph. abs., absolute lymphocytes count; Lymph.%, lymphocytes percentage; RBC, red blood cells; Hb, hemoglobin. * indicates significant p value (p<0.05) as indicated by t-test. Significant p values (<0.05) are indicated by bold text.

### 3.2 A2M levels in patients and NC subjects – association with CLL stage and mutation status

**Figure 1:**
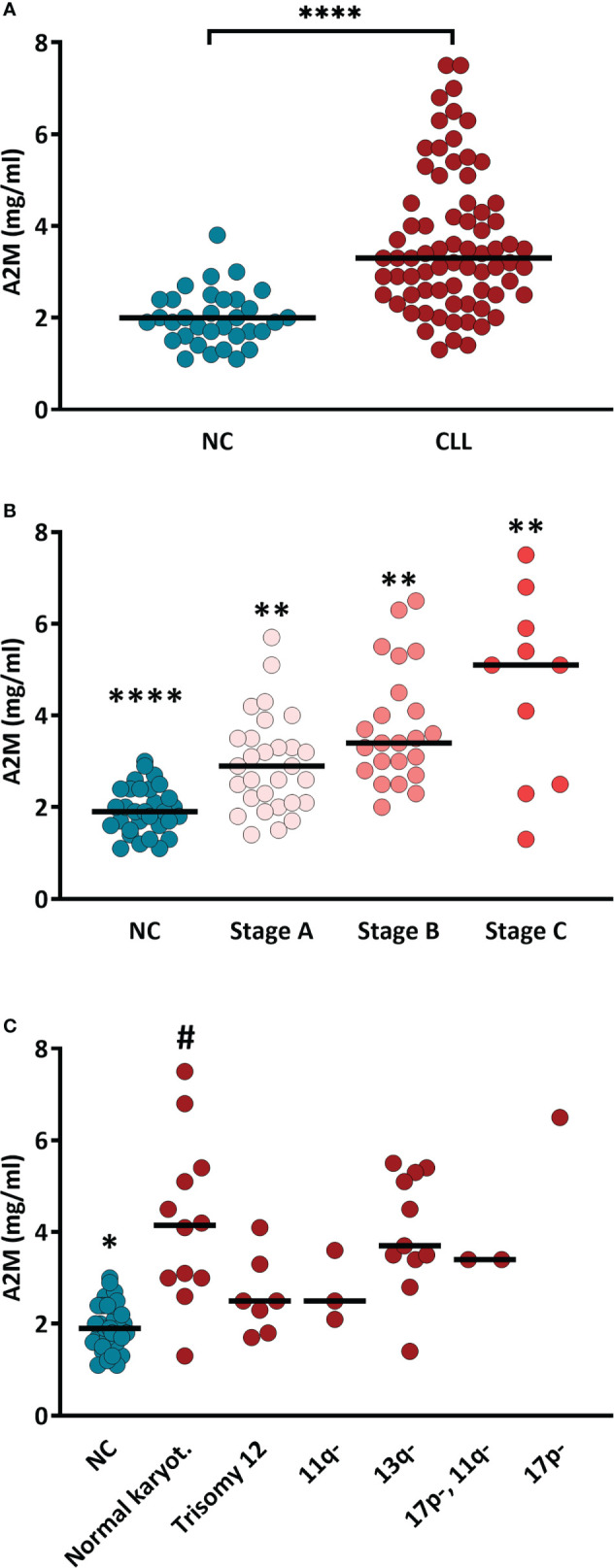
Serum A2M levels in CLL patients and NC subjects. **(A)** A2M levels were determined (using ELISA) in sera of CLL patients (n = 65) and NC subjects (n = 30). **** indicates p<0.0001 by t-test. **(B)** A2M levels in sera of CLL patients divided according to the CLL stage (Binet staging; stage A: n=29, stage B: n=23, stage C: n=12). **** and ** indicate p<0.0001 and p<0.02, respectively, vs. each other group. **(C)** Serum A2M levels in CLL patients divided according to the mutation associated with CLL. * indicates p<0.05 vs. each other group, # indicates p<0.05 vs. the NCs and vs. the CLL groups with 11q- and trisomy 12, as determined by the Mann-Whitney test.

The serum levels of A2M were significantly higher (p<0.0001) in patients vs. NCs, showing values of 3.62 ± 0.22 and 1.97 ± 0.10 mg/ml, respectively (**Figure 1A**). Within the CLL group, A2M levels did not correlate with age, and was similar in males and females (**Supplementary Figure S1**).

When the patients cohort was divided into subgroups according to the disease stage (Binet staging), the serum A2M levels showed clear association with the disease stage, as can be seen in the gradual increase in A2M values along with the CLL stages (**Figure 1B**). The values of A2M in patients at stage A, B and C were 2.86 ± 0.18, 3.76 ± 0.26 and 5.61 ± 0.83 mg/ml, respectively, and were significantly different (p<0.01) between the groups (**Figure 1B**).

Genetic analysis of CLL-associated genes was available from 36 out of the 65 patients included in the study. This analysis is not a routine test in the care of CLL patients with indolent disease, and is usually performed when the disease advances and the patient may face intervention. Therefore, the distribution of CLL stages in these 36 patients differed from the stage distribution in the original cohort, with relatively decreased proportion of patients in stage A (only 19% vs. 44% in the 65 patients cohort) and increased proportion of patients at stages B (53% vs. 38%) and C (28% vs. only 18%).

Among these 36 patients with available genetic data, 12 (33%) had normal karyotype, 7 (19%) had trisomy 12, 3 (8%) had 11q deletion, 11 (31%) had 13q deletion, and only one patient (3%) had 17p deletion and 2 patients (6%) had both 17p and 11q deletions. When A2M levels were compared between the CLL subgroups divided according to the genetic abnormalities (**Figure 1C**), high levels (4.2 ± 0.5 mg/ml) were found in patients with normal karyotype, and lower A2M levels, although significantly higher compared with NCs, were found in patients with Trisomy 12 (2.6 ± 0.32 mg/ml) or with deletions of the 11q or 13q (2.9 and 4.0 mg/ml, respectively). The high A2M level in the only patient with the 17p deletion (6.5 mg/ml), should be further verified in order to establish this observation, particularly since 17p deletion, as a sole abnormality is associated with poor prognosis: short survival and the shortest treatment-free interval (13). The prognosis for patients with normal karyotype, however, is better relative to CLL patients with one or more genetic abnormalities (13).

Overall, the data in **Figure 1** show that A2M levels are increased in CLL and are associated with indicators for aggravation of the patient’s condition (CLL stage), but it is unclear if they are also associated with genetic indicators for poor prognosis.

### 3.3 Association of A2M levels with hematological parameters

The association of serum A2M levels with hematological parameters was analyzed and the resulting correlation data is given in **Table 2**. The graph for each correlation analysis is given in **Supplementary Figure S2**. The data showed significant positive correlations with WBC, lymphocytes numbers and % of CD38+ B-cells (in patients with CD38+ staining), and significant negative correlations with RBC, platelet counts and hemoglobin levels (**Table 2**). The data indicate, again, that A2M levels in CLL are associated with indicators for disease severity, including high WBC and lymphocyte counts, and low RBC, Hb and platelet counts.

**Table 2:** Association of serum A2M levels with hematological parameters.

Clinical parameters	R value	p value
WBC	0.29	**0.02**
Lymph. abs.	0.42	**0.004**
Lymph. %	0.09	0.056 (ns)
Platelets	-0.48	**0.0001**
RBC	-0.42	**0.0045**
Hb	-0.44	**0.0004**
CD38+ %	0.56	**0.024**

The results of linear correlation analysis between serum A2M levels and hematological parameters are shown. WBC, white blood cells; Lymph. abs., absolute lymphocytes count; Lymph%., lymphocytes percentage; RBC, red blood cells; Hb, hemoglobin; % CD38+, percentage of CD38 positive B-lymphocytes; ns, non-significant p value. P value <0.05 was considered significant. Significant p values (<0.05) are indicated by bold text.

### 3.4 Association of A2M levels with biochemical parameters

The associations of serum A2M levels with biochemical parameters are provided in **Table 3**. The graph for each correlation analysis is given in **Supplementary Figure S3**. The data showed significant positive correlations with alkaline phosphatase (ALP), aspartate aminotransferase (AST), glucose and b2M levels, and a significant negative correlation with HDL. Among the biochemical parameters that were used for correlation analyses, b2M and abnormal liver function are known to be associated with the CLL staging (14). Thus, the data in **Table 3** support the association of A2M levels with indicators for aggravation of the CLL disease.

**Table 3:** Association of serum A2M levels with biochemical parameters.

Clinical parameters	R value	p value
ALP	0.53	**0.0007**
AST	0.43	**0.0074**
ALT	-0.22	0.180 (ns)
Glucose	0.47	**0.0026**
HDL	-0.47	**0.0018**
Non-HDL Chol.	0.083	0.63 (ns)
LDL	0.007	0.99 (ns)
Cholesterol	-0.104	0.52 (ns)
Triglycerides	0.21	0.186 (ns)
b2M	0.62	**0.0033**

The results of linear correlation analysis between A2M levels and biochemical parameters are shown. ALP, alkaline phosphatase; AST, aspartate aminotransferase; ALT, alanine transaminase; HDL, high density lipoprotein; LDL, low-density lipoprotein; non-HDL Chol., Non-HDL Cholesterol; B2M, beta-2-microglobulin; ns, non-significant p value. P value <0.05 was considered significant.Significant p values (<0.05) are indicated by bold text.

### 3.5 Association of A2M levels with complement-related parameters

**Figure 2:**
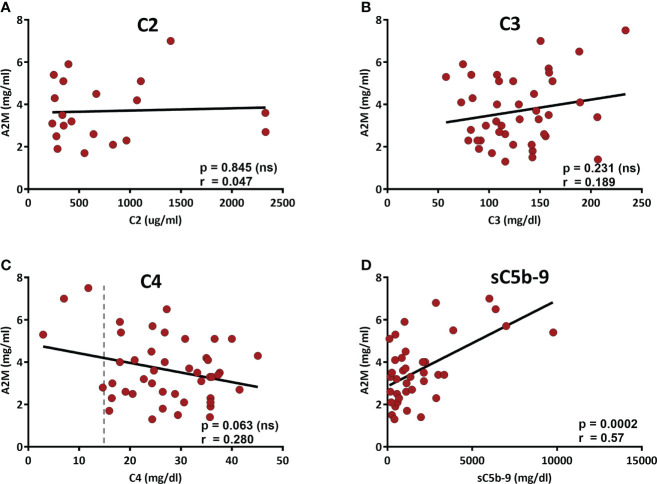
Correlation of the serum A2M levels with complement components. The levels of the complement components C2-C4 and the complement activity marker sC5b-9 (the final product of complement activation) were measured in patients’ sera. A2M levels were correlated with C2 **(A)**, C3 **(B)**, C4 **(C)** and sC5b-9 **(D)**. The dashed line indicates 15 mg/dL, which is the lower level of the normal range (15-57 mg/dL).

We recently showed the involvement of A2M in the formation of cell-free aggregates that act as activators of the CP (4). Therefore, the correlation of serum A2M levels with complement-related parameters was studied. The correlations with the levels of C2 and C4, that are essential components of the CP and can presumably be exhausted by chronic CP activation, were studied, as well as the correlation with the levels of C3. Also, the baseline levels of sC5b-9, the final product of complement activation and a marker of complement activity, are associated with chronic activation of the CP (15), and therefore its correlation with the A2M levels were assessed as well. The correlation data, presented in **Figure 2**, did not show significant correlation with either C2, C3 or C4 (**Figures 2A–C**). However, a significant positive correlation was found in the CLL patients with the baseline levels of sC5b-9 (**Figure 2D**), supporting a link between A2M levels and chronic activation of the CP.

Interestingly, although the correlation of A2M and C4 did not reach a significant p value (p=0.063), the data in **Figure 2C** shows that among the 4 patients with C4 values below the normal range (i.e. <15 mg/dL), 3 patients had very high A2M levels, clearly above (>twice) the normal A2M average (which is 1.97 ± 0.10 mg/ml). Alas, examination of the A2M values in the subgroup of 4 patients with sub-normal C4 values vs the rest of the CLL patients indicated a p value of 0.052. Yet, this result suggests that analysis of a larger group of patients with sub-normal C4 values may eventually indicate significant differences that can further support the link between A2M levels and chronic activation of the CP. This assumption is supported by a correlation analysis of A2M with C2-C4, which was performed in a larger cohort, including both CLL and NC subjects, and showed significant correlation with the levels of C4, but not with C2 or C3 (**Supplementary Figure S4**).

Altogether, the data indicate that A2M levels in CLL are not only associated with the disease severity, but are also significantly associated with sC5b-9, which is a marker of chronic complement activation.

### 3.6 Production of A2M in B-lymphocytes

**Figure 3:**
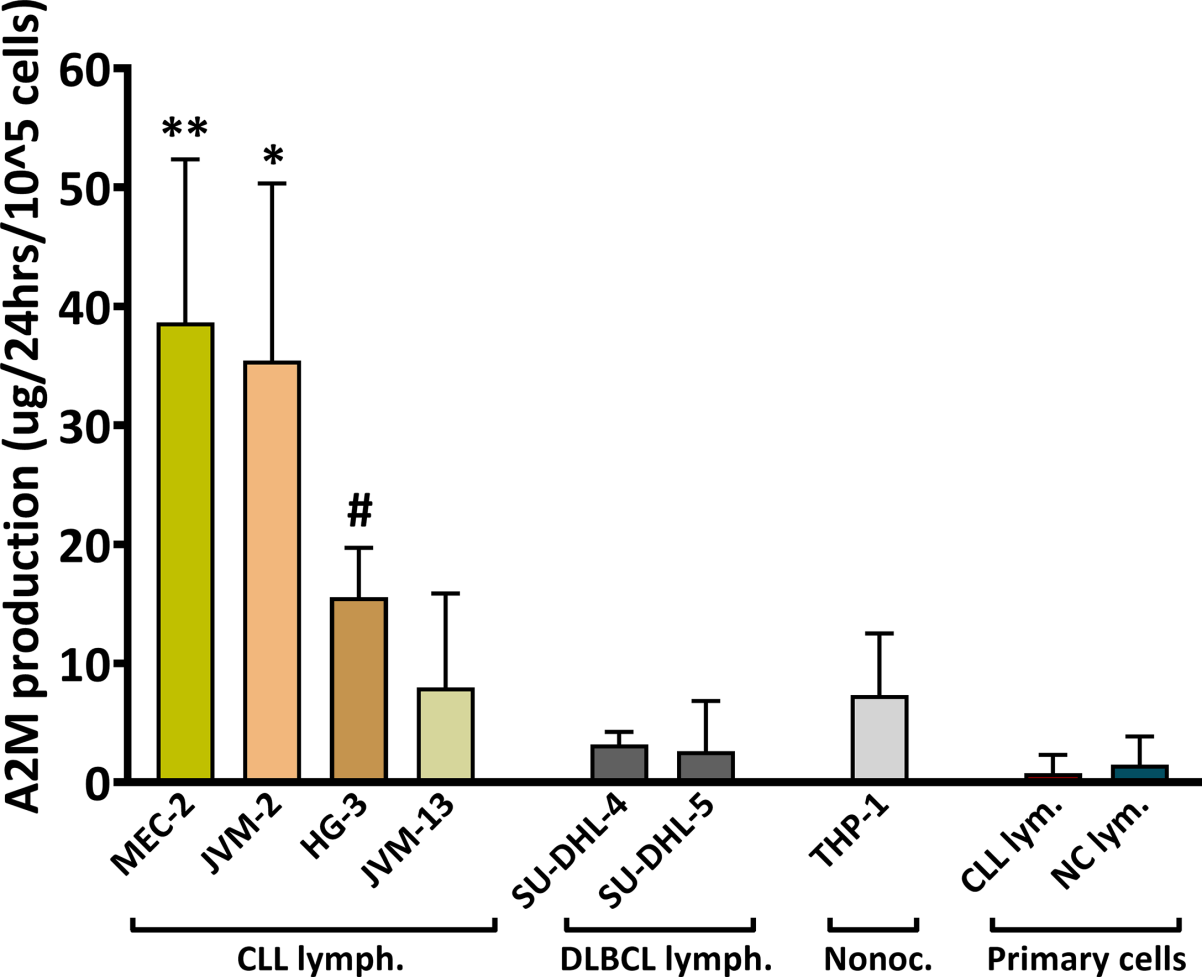
A2M production in B-Cells. The production of A2M was studied in the CLL cell lines MEC-2, JVM-2, JVM-13, HG-3, in the non-CLL B-lymphocytic cell lines SU-DHL-4 and SU-DHL-5, and in one monocytic cell line, THP-1. The assay was repeated ≥3 times for each cell line (n = 5 for MEC-2 and HG-3, n = 4 for JVM-13). A2M production was also studied in B-lymphocytes (primary cells) separated from peripheral blood of CLL patients (CLL lym.; n = 4) and NC subjects (NC lym.; n = 4). Cells were incubated in triplicates at a density of 20,000-40,000 cells per 100µl in the appropriate medium for each line, for 12-72 hrs and the ΔA2M levels were calculated for 100,000 cells for 24 hrs. ** indicates significant p values < 0.01 compared to each of the other cell lines, except for JVM-2; * indicates significant p values < 0.05 compared to each of the other cell lines, except for MEC-2; # indicates significant p values (p< 0.05) compared to each of the other cell lines, except for JVM-13 and THP-1.

A2M is produced primarily in the liver and lungs, but local production by macrophages, fibroblasts, and epithelial cells was also reported (5). The increased levels of A2M in CLL serum and the association with WBC and particularly with lymphocytes counts, encouraged us to explore the possibility that in CLL patients, A2M levels may be increased in the circulation due to local production by B-lymphocytes. This was first examined in the CLL cell lines MEC-2, JVM-2, JVM-13 and HG-3 that were compared with the non-CLL B-lymphocytic cell lines SU-DHL-4 and SU-DHL-5, and with a monocytic cell line, THP-1 (**Figure 3**). When compared with non-CLL or monocytic lines, the data clearly indicated higher production of A2M in the CLL B-lymphocytic cell lines that were examined, particularly in the MEC-2 and JVM-2 lines (**Figure 3**). In one CLL cell line, the JVM-13, the increase in A2M production was not as pronounced as in the other CLL lines.

The A2M production was further studied in primary B-lymphocytes, isolated from peripheral blood of CLL patients and NC subjects. These experiments utilized separated B-cells with a ~99% purity (validated by flow-cytometry; showing 1.1 ± 0.7% and 1.1 ± 0.5% of non-B-cells for separations from CLL and NC, respectively), and were performed in order to verify the findings in primary cells, as well as to examine A2M production by B-lymphocytes from NC subjects. The results indicated nearly no A2M production by primary B-lymphocytes from both patients and NC subjects (**Figure 3**). Clearly, the production of A2M by primary B-lymphocytes was lower than its production by the CLL cell lines.

### 3.7 Production of A2M in B-lymphocytes depends on serum factor/s

**Figure 4:**
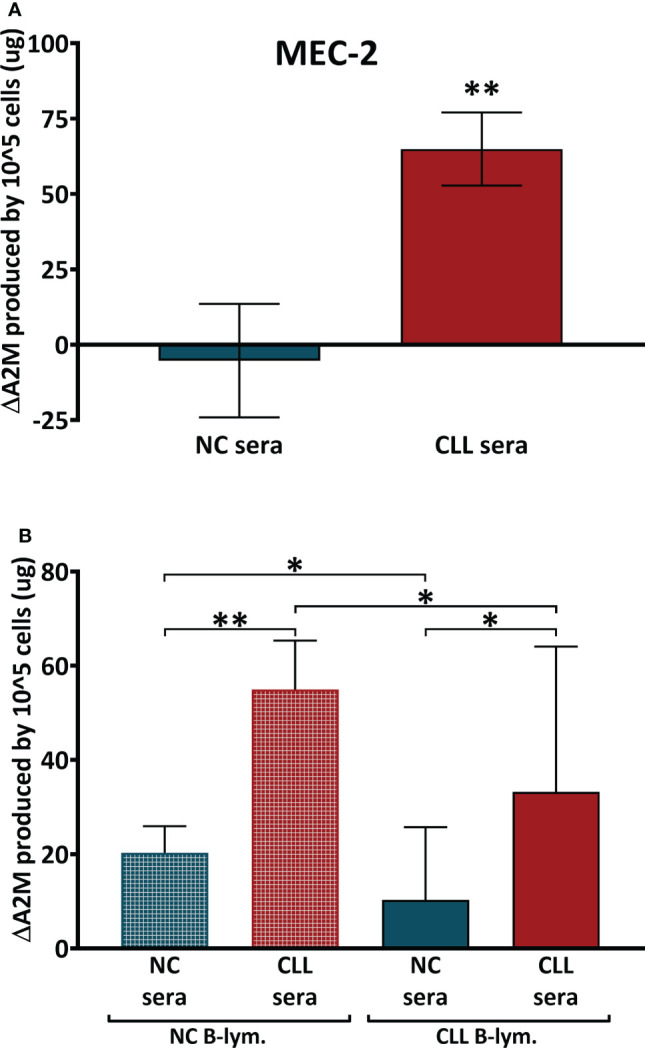
A2M production in B-Cells after incubation with sera. **(A)** The production of A2M was studied in the CLL cell line MEC-2 (n = 25) and in primary B-lymphocytes **(B)** that were separated from peripheral blood of CLL patients (CLL B-lym.) and NC subjects (NC B-lym.);. Cells were incubated with NC sera (n = 10 with MEC-2 cells, n=11 with NC-lym. and n = 12 with CLL lym.) or with CLL sera (n = 15 with MEC-2 cells, n = 12 with NC-lym. and n = 11 with CLL lym.), A2M levels were measured and the ΔA2M levels were calculated for 100,000 cells per 24 hrs. ** indicates significant p values < 0.01 and * indicates significant p values < 0.05.

The data from CLL cell lines indicated that B-lymphocytes have the ability to produce A2M, so we next focused on initial characterization of the factor/s that may cause or influence A2M production in CLL, by studying whether A2M production depends on the cells’ surroundings. This was examined in primary B-lymphocytes from peripheral blood and in the CLL cell line MEC-2, since MEC-2 showed the highest A2M production rate among the four CLL cell lines that were examined. In these experiments MEC-2 cells were incubated for 12 hrs with NC or CLL sera and the A2M levels were measured in the culture medium. The production of A2M by the cells was calculated as the change in A2M levels (ΔA2M) after subtraction of the A2M contributed by the added serum, and the levels self-produced by the cells (i.e. without any added serum). The average ΔA2M levels after incubation of MEC-2 cells with NC sera was -5 ± 19 µg, indicating no change or a very small decrease in A2M levels, while incubation with CLL sera resulted in significantly (p=0.0011) higher ΔA2M, with an average of 65 ± 12 µg (**Figure 4A**).

In the next set of experiments, primary B-lymphocytes were isolated, incubated with identical sera samples (CLL and NC sera) for 24 hrs, and the A2M levels in the incubation media were quantified. The data in **Figure 4B** showed increases in A2M production after incubation of the primary cells with sera. However, the increases in A2M production was significantly greater when cells were incubated with patients’ sera, relative to incubations with NC sera. This was observed in both NC and patients’ lymphocytes (**Figure 4B**). Interestingly, the responsiveness of CLL cells to the sera was lower than that of NC cells, so that upon incubation, CLL cells were unable to increase their A2M production to the extent seen with NC cells (**Figure 4B**).

Altogether, these data suggest that the increased A2M production by CLL B**-**lymphocytes probably depends both on factor/s that exist in the CLL serum (such as IL-6-type cytokines), as well as on the capability of the cells to respond to these factors, an ability that appears to exist in both CLL and NC B-lymphocytes.

## 4 Discussion

This study shows for the first time that the serum levels of A2M in CLL are significantly increased compared to normal control subjects, and are correlated with the disease stage, with various biochemical and hematological parameters associated with the severity of the disease, and with chronic complement activation. Moreover, increased serum levels of A2M may be, at least partially, due to A2M production by the malignant B-lymphocytes.

Our previous studies that focused on the chronic CP activation in CLL included characterization of the cell-free IgG-aggregates that exist in CLL serum and act as a CP activator (4, 16). In the initiation of CP activation, IgG-hexamers bind C1, the first component of the CP, and activate a cascade of events until C5b-9 (MAC), the final complement product, is generated. IgG-aggregates are assembled in the form of hexamers after an antigen binds to each IgG monomer and non-covalent Fc-Fc interactions occur (17). In CLL patients, A2M was found to be the antigen causing the hexamerization and the assembly of the cell-free IgG-aggregates (4). The factors that control the cell-free hexamerization process in CLL plasma are not yet discovered, however one can assume that this process requires the existence of anti-A2M IgG molecules, along with available A2M, hypothetically at levels that are increased above normal, at least for one of these two factors. The present study demonstrates one of these conditions in CLL patients, showing A2M levels that are increased by 84% in the entire CLL patient’s cohort, and by up to 185% in patients with stage C CLL.

The correlation analyses with different hematological parameters also suggest the association of A2M levels with the CLL disease severity, as shown by the correlations with the established CLL parameters (18): RBC, platelet counts, lymphocyte counts and Hb levels. Moreover, this observation is also supported by the correlation data of A2M with various biochemical parameters that were shown to increase with CLL severity, such as abnormal liver function tests (LFT) (14), b2M (19), blood glucose levels/Diabetes mellitus (DM, 20), and HDL that is decreased in CLL and particularly in the advanced CLL stages (12). Normal karyotype is a not an indicator of poor prognosis for CLL patients (13), unlike the immunoglobulin gene heavy-chain variable region (IGHV) gene, where absence of mutations (unmutated IGHV) is associated with poor outcomes (21, 22). The increased level of A2M in patients with normal karyotype compared to patients with genetic aberrations should be further investigated in order to clarify whether serum A2M levels are associated with the disease prognosis.

Since A2M is produced by several human tissues, the excess A2M levels in the circulation of CLL patients may originate from various cells/organs. In this study, we have evaluated the possibility that the B-lymphocytes in CLL may produce A2M and thus contribute to the increase in serum A2M levels. We demonstrated that circulating B-lymphocytes have the ability to produce significant amounts of A2M in response to factors that exist in CLL serum. After incubation with sera, the A2M production capacity of CLL-B-lymphocytes was somewhat decreased (~40%) compared with NC-B-lymphocytes. However, it is reasonable to assume that the huge numbers of B-lymphocytes in CLL circulation, 20 times higher than in NC (**Table 1**), can certainly overcome the 40% difference in production capacity of the B-cells and eventually result in significantly increased A2M levels in CLL serum. The significant correlation of A2M levels with the lymphocyte counts also supports this possibility.

A2M production in primary B-lymphocytes exposed to CLL sera or NC sera showed an increase of 23 µg A2M per 10^5^ cells (33 vs 10 µg A2M/10^5^ cells, for incubation with CLL vs. NC sera, respectively). Based on these data, and on the essential condition of at least 5000 monoclonal lymphocytes per µl that is requisite to fulfill the criteria for CLL diagnosis, the minimal amount of excess A2M produced by the B-lymphocytes was calculated to be 1.15 mg/ml. This value is in very good agreement with the data showing an increase of ~1.6 mg/ml in A2M values in CLL vs. NC subjects (from 1.97 ± 0.10 to 3.6 ± 0.22 mg/ml in NC and CLL subjects, respectively). For the majority of CLL patients, the numbers of monoclonal lymphocytes per µl are considerably above 5000 and normally reach ~67% (56-78%) of the total lymphocyte count (23), hence the amounts of A2M produced are presumably increased accordingly. This is particularly evident at advanced CLL stages. Although some of the A2M molecules bind to T-lymphocytes and to the B-lymphocytes *via* the CD91 receptor (24) and the GRP78 receptor (4, 25, 26), respectively, the increased amounts of A2M produced, yield serum levels that correlate very well with the lymphocytes’ counts in the circulation. In the experiments assessing A2M production by B-cells, the potential binding to the CD91 receptor on T-lymphocytes is irrelevant, since these experiments were performed using only B-cells (in case of cell lines) or 99% pure primary B-cells preparations. The potential binding to the GRP78 receptor exists, and definitely deserves further studies. If some of the produced A2M molecules bind to the GRP78 receptor, it would mean that the actual amount of A2M produced by the B-cells is higher than the measured values.

The study also showed that most cell-lines of CLL B-lymphocytes that were analyzed are capable of producing A2M even in absence of the CLL-serum-factors. Altogether, the data suggest that the excess A2M in CLL patients’ sera may be, at least partly, due to production by the numerous B-lymphocytes in the circulation.

Study limitations: Although the study was conducted on an adequate size of CLL patient’s population, karyotype information was available only from a limited number of patients, only 36 out of the 65 patients included in the study. As a result, the distribution of CLL stages in these 36 patients differed from the stage distribution in the original cohort and may not be representative of the stage distribution, or the mutational status in the entire patient’s cohort. Also, due to this limitation, some mutations were only observed in one (17p deletion) or two (17p and 11q deletions) patients, making statistical analysis irrelevant for some CLL subgroups categorized by these specific mutations. Thus, this data set should be analyzed with caution, and the apparently high level of A2M found in the only patient with 17p deletion, for example, should be further validated in additional patients with a similar karyotype.

Also, although the study was not limited only to descriptive data and correlation indices, and the mechanisms that affect A2M production were assessed, we have not yet fully described the CLL-serum-factor/s that trigger A2M production in B-lymphocytes. The data available from studies in hepatocytes indicate the involvement of cytokines that are released during inflammatory processes, with IL-1- and IL-6-type cytokines as leading regulators of the acute phase response (27). These cytokines show additive, inhibitory, or synergistic regulatory effects on A2M expression. A2M production by B-cells has not yet been studied, however autocrine IL‐6 production by CLL B-cells has been described (28), and was shown to be associated with poor clinical outcome in CLL (29) due to IL‐6‐mediated survival mechanisms that provide resistance of CLL cells to chemotherapy. In primary CLL B-cells, IL‐6 activated both STAT3 and NF‐κB, similar to the cascade described in hepatocytes (29), and we believe that this IL-6 mediated control mechanism can potentially control A2M production in CLL B-lymphocytes.

From a translational perspective, we believe that the results may be useful for following the severity of the CLL disease, before and potentially during immunotherapy treatments. The data may also be used for developing advanced immunotherapy treatments, combined with inhibitors of excess A2M production (such as IL-1β or an IL-1β-agonist), and thus the IgG-hexamerization and the resulting chronic CP activation may be down-regulated, helping the complement system attain maximal capacity. Such A2M inhibitors may improve the patients’ complement system activity against infections, as well as their response to immunotherapy.

## Data availability statement

The raw data supporting the conclusions of this article will be made available by the authors, without undue reservation.

## Ethics statement

The studies involving human participants were reviewed and approved by Institutional Review Board of Galilee Medical Center, Nahariya, Israel. The patients/participants provided their written informed consent to participate in this study.

## Author contributions

RM: conceptualization, data curation, data analysis, methodology, project administration, supervision, validation, writing – original draft, writing – review and editing. LM: data curation, methodology. EG: data curation, methodology. GS: resources, review and editing. TT: resources. AA: resources. MS: methodology. LS: resources. MB: project administration, supervision. AB: supervision, resources, project administration, methodology, funding, conceptualization, review and editing. All authors contributed to the article and approved the submitted version.

## Funding

The study was funded by the the Health Corporation of Galilee Medical Center, Nahariya, Israel.

## Acknowledgments

The authors thank Prof. Chaim Putterman and Dr. Adi Litmanovich for their help in editing and proofreading of the manuscript.

## Conflict of interest

The authors declare that the research was conducted in the absence of any commercial or financial relationships that could be construed as a potential conflict of interest.

## Publisher’s note

All claims expressed in this article are solely those of the authors and do not necessarily represent those of their affiliated organizations, or those of the publisher, the editors and the reviewers. Any product that may be evaluated in this article, or claim that may be made by its manufacturer, is not guaranteed or endorsed by the publisher.

## Supplementary material

Supplementary Figure 1 Association of serum A2M levels with gender and age.The levels of A2M in sera of male and female **(A)** and the correlation between serum A2M and age **(B)** were studied in the CLL patients. Abbreviations: ns: non-significant p value. P value <0.05 was considered significant.

Supplementary Figure 2 Association of serum A2M levels with hematological parameters.The results of linear correlation analysis between serum A2M levels and hematological parameters are shown. WBC: white blood cells; Hb: hemoglobin; CD38(%): percentage of CD38 positive B-lymphocytes; RBC, red blood cells; Lymph.#., absolute lymphocytes count; Lymph%., lymphocytes percentage; ns, non-significant p value. P value <0.05 was considered significant.

Supplementary Figure 3 Association of serum A2M levels with biochemical parameters.The results of linear correlation analysis between A2M levels and biochemical parameters are shown. ALP, alkaline phosphatase; AST, aspartate aminotransferase; ALT, alanine transaminase; HDL, high density lipoprotein; LDL, low-density lipoprotein; non-HDL Chol., Non-HDL Cholesterol; b2M, beta-2-microglobulin; ns, non-significant p value. P value <0.05 was considered significant.

Supplementary Figure 4 Correlation of serum A2M levels with complement components in CLL and NC. The levels of the complement components C2-C4 were measured in sera of CLL patients and NC subjects. A2M levels were correlated with C2 **(A)**, C3 **(B)** and C4 **(C)**. The dashed line indicates 15 mg/dL, which is the lower level of the normal range (15-57 mg/dL). ● indicate CLL patients and ○ indicate NC subjects.
