# Supplementary figures and images for: Increased serum level of alpha-2 macroglobulin and its production by B-lymphocytes in chronic lymphocytic leukemia
Source: Front Immunol. 2022 Sep 2;13:953644. doi: 10.3389/fimmu.2022.953644 (PMC9478581; doi:10.3389/fimmu.2022.953644)

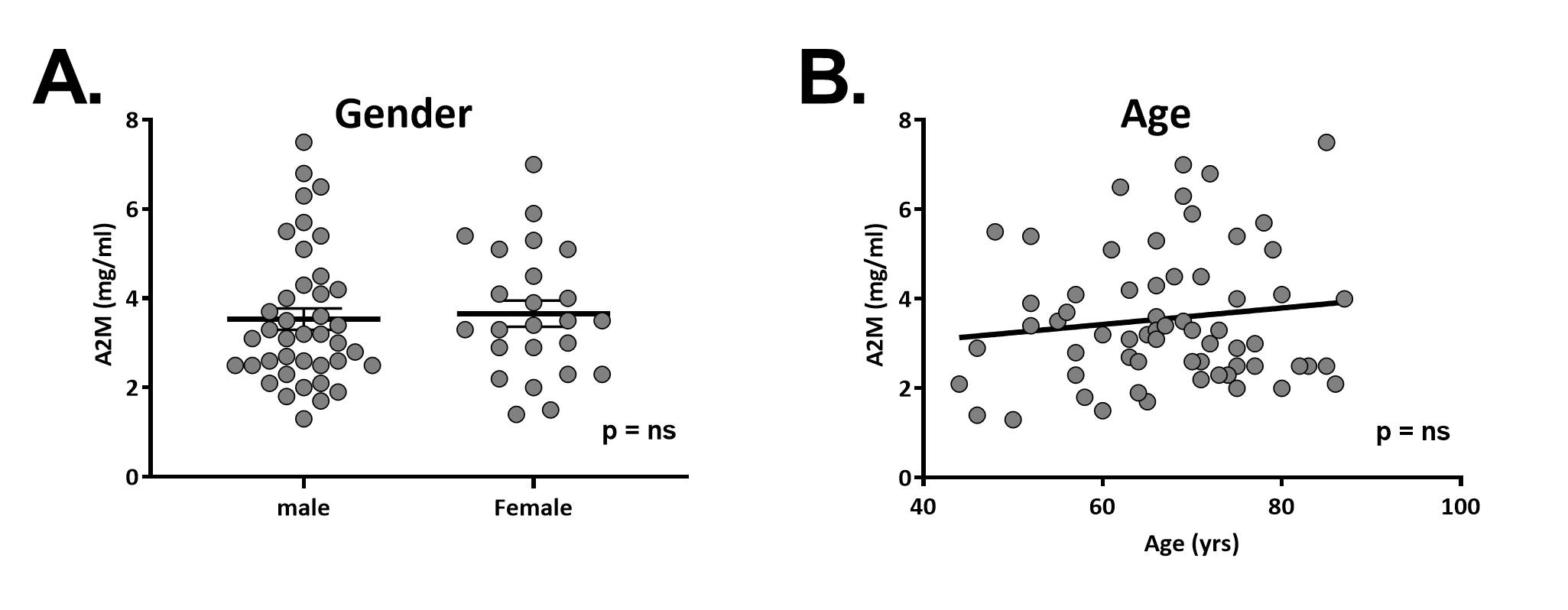

Supplement: Supplementary Figure 1 — Association of serum A2M levels with gender and age.The levels of A2M in sera of male and female (A) and the correlation between serum A2M and age (B) were studied in the CLL patients. Abbreviations: ns: non-significant p value. P value <0.05 was considered significant. [file Image_1.tif]

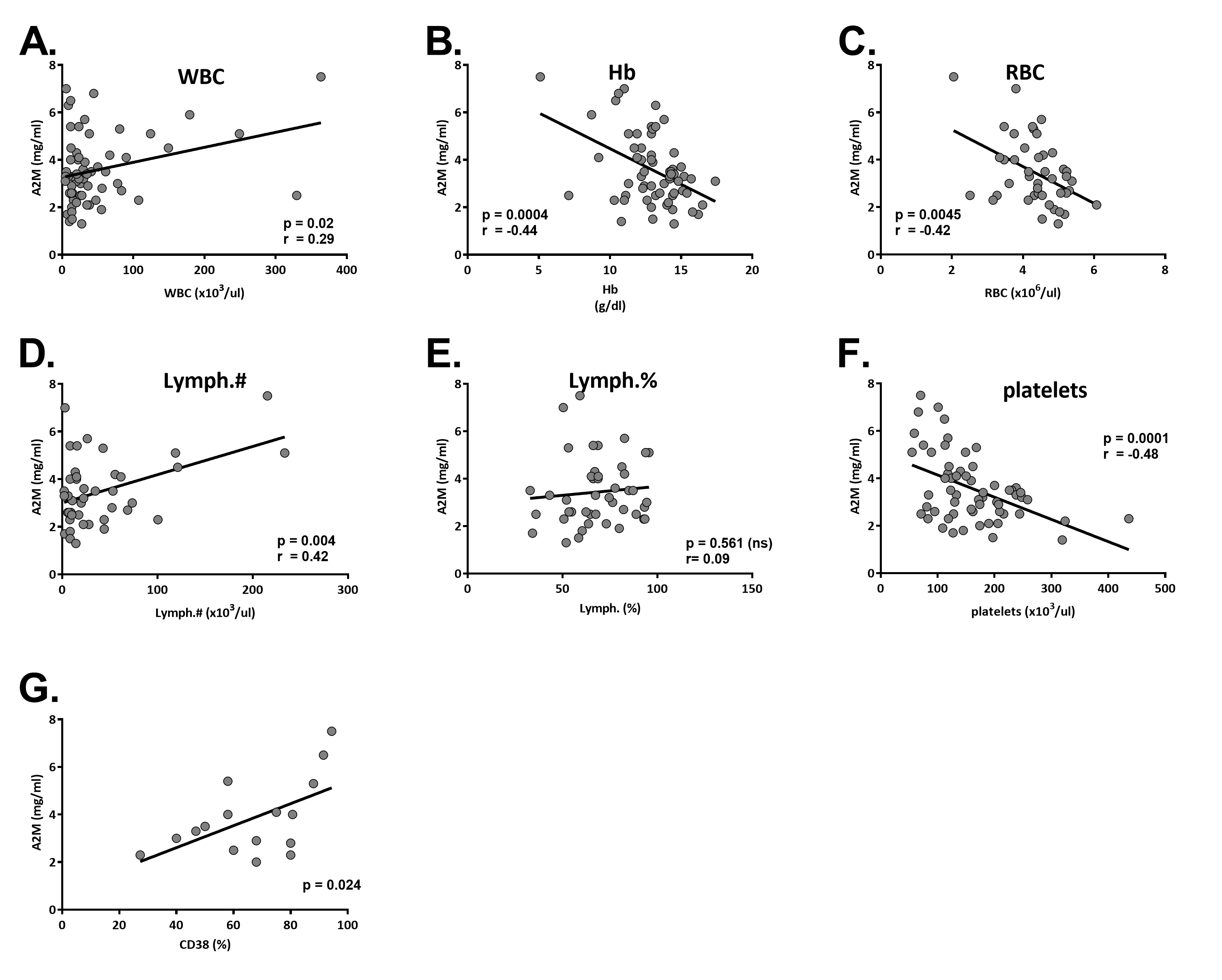

Supplement: Supplementary Figure 2 — Association of serum A2M levels with hematological parameters.The results of linear correlation analysis between serum A2M levels and hematological parameters are shown. WBC: white blood cells; Hb: hemoglobin; CD38(%): percentage of CD38 positive B-lymphocytes; RBC, red blood cells; Lymph.#., absolute lymphocytes count; Lymph%., lymphocytes percentage; ns, non-significant p value. P value <0.05 was considered significant. [file Image_2.tif]

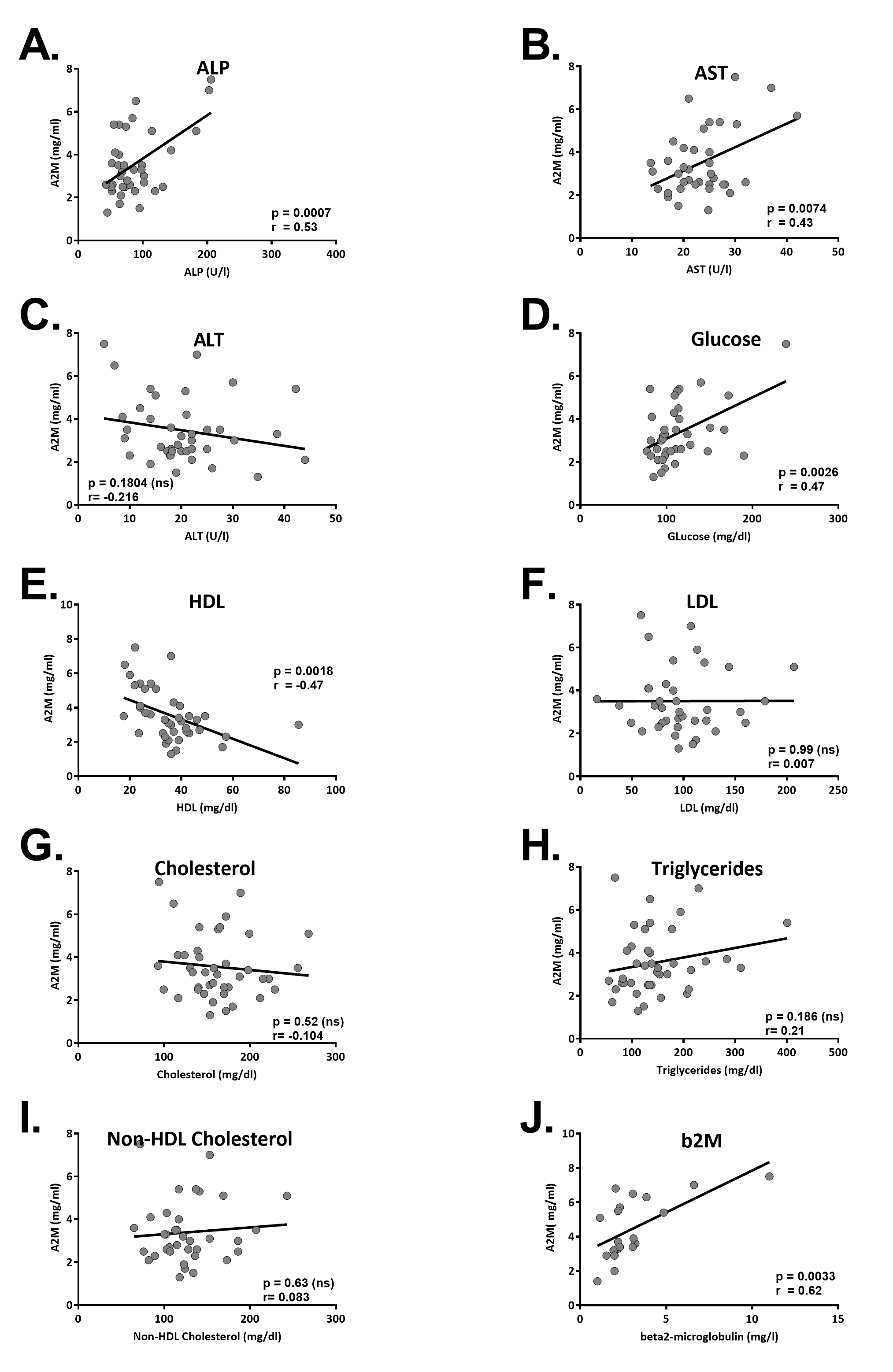

Supplement: Supplementary Figure 3 — Association of serum A2M levels with biochemical parameters.The results of linear correlation analysis between A2M levels and biochemical parameters are shown. ALP, alkaline phosphatase; AST, aspartate aminotransferase; ALT, alanine transaminase; HDL, high density lipoprotein; LDL, low-density lipoprotein; non-HDL Chol., Non-HDL Cholesterol; b2M, beta-2-microglobulin; ns, non-significant p value. P value <0.05 was considered significant. [file Image_3.tif]

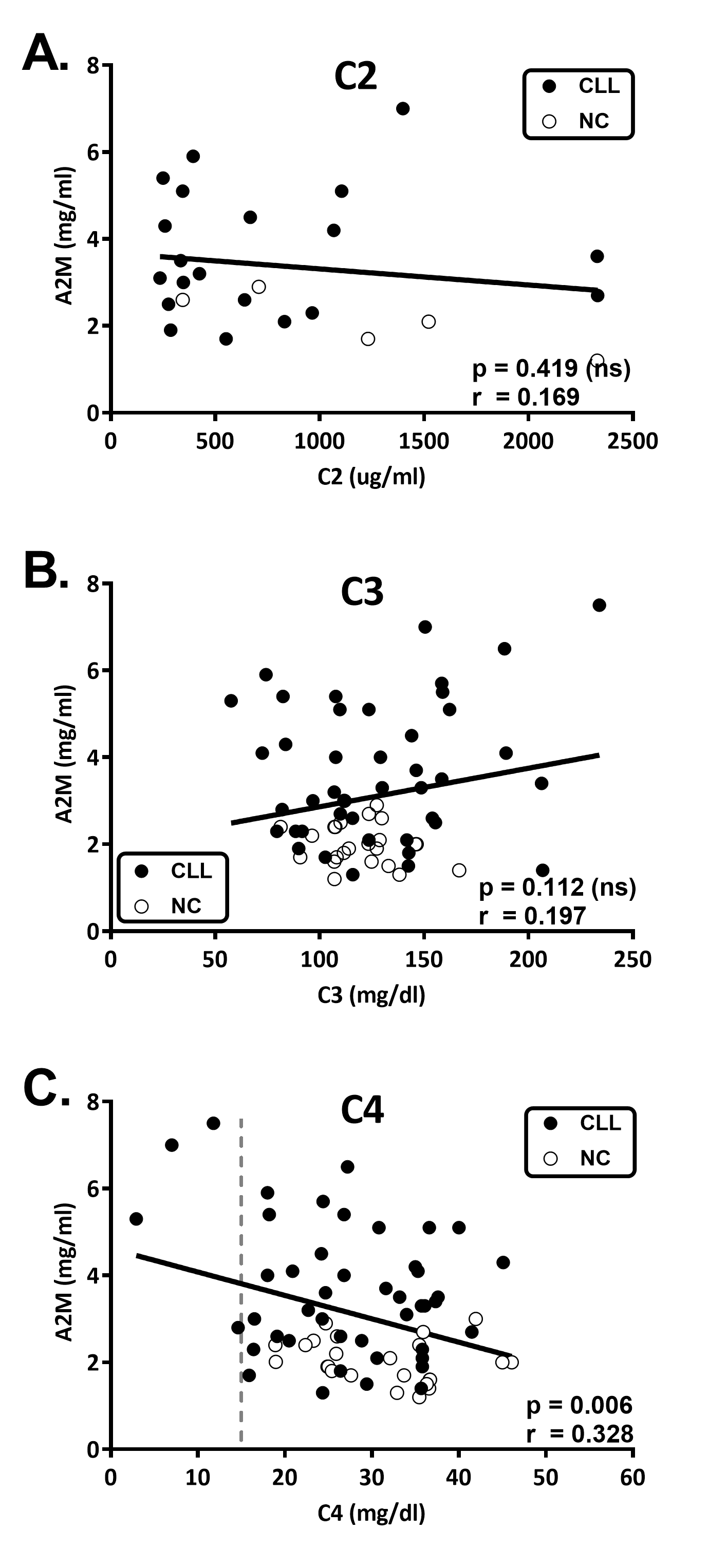

Supplement: Supplementary Figure 4 — Correlation of serum A2M levels with complement components in CLL and NC. The levels of the complement components C2-C4 were measured in sera of CLL patients and NC subjects. A2M levels were correlated with C2 (A), C3 (B) and C4 (C). The dashed line indicates 15 mg/dL, which is the lower level of the normal range (15-57 mg/dL). ● indicate CLL patients and ○ indicate NC subjects. [file Image_4.tif]
